# Supplementary material for: Effectiveness and Safety of Inactivated SARS-CoV-2 Vaccine (BBIBP-CorV) among Healthcare Workers: A Seven-Month Follow-Up Study at Fifteen Central Hospitals
Source: Vaccines (Basel). 2023 Apr 24;11(5):892. doi: 10.3390/vaccines11050892 (PMC10224440; doi:10.3390/vaccines11050892)
Supplement: Supplementary file 1 [file vaccines-11-00892-s001.zip › vaccines-2352133-supplementary.pdf]

Table S1. Baseline characteristics of healthcare workers at the first interview

Item	COVID vaccination status			P-value
	**Unvaccinated (N=898)**	**Vaccinated (N=330)**	**Total (N=1228)**	
**Age**				**< 0.001**
Median (IQR)	30.0 (26.0, 37.0)	35.0 (29.0, 42.0)	31.0 (27.0, 39.0)	
**Body mass index (BMI)**				**< 0.001**
Median (IQR)	26.2 (24.1, 28.7)	27.5 (25.5, 30.9)	26.6 (24.4, 29.3)	
**Healthcare Profession**				**< 0.001**
Dentist	15 (1.7%)	1 (0.3%)	16 (1.3%)	
Nurse	569 (63.4%)	71 (21.5%)	640 (52.1%)	
Pharmacist	117 (13.0%)	132 (40.0%)	249 (20.3%)	
Physician	127 (14.1%)	79 (23.9%)	206 (16.8%)	
Technician	70 (7.8%)	47 (14.2%)	117 (9.5%)	
**Healthcare workplace at hospital**				**< 0.001**
Clinic	104 (11.6%)	21 (6.4%)	125 (10.2%)	
ER	78 (8.7%)	7 (2.1%)	85 (6.9%)	
Ward	202 (22.5%)	51 (15.5%)	253 (20.6%)	
ICU	313 (34.9%)	65 (19.7%)	378 (30.8%)	
Inpatient pharmacy	72 (8.0%)	80 (24.2%)	152 (12.4%)	
Outpatient pharmacy	14 (1.6%)	22 (6.7%)	36 (2.9%)	
Laboratory	35 (3.9%)	12 (3.6%)	47 (3.8%)	
Others	80 (8.9%)	72 (21.8%)	152 (12.4%)	
**Hospital type**				**< 0.001**
Chest	515 (57.3%)	77 (23.3%)	592 (48.2%)	
Fever	97 (10.8%)	111 (33.6%)	208 (16.9%)	
Isolation	286 (31.8%)	142 (43.0%)	428 (34.9%)	
**Governorate**				**< 0.001**
Alexandria	222 (24.7%)	59 (17.9%)	281 (22.9%)	
Assuit	190 (21.2%)	36 (10.9%)	226 (18.4%)	
Cairo	156 (17.4%)	19 (5.8%)	175 (14.3%)	
Kafr-Elsheikh	260 (29.0%)	100 (30.3%)	360 (29.3%)	
Sharqia	70 (7.8%)	116 (35.2%)	186 (15.1%)	
**Obesity BMI > 35**				**< 0.001**
No	865 (96.3%)	296 (89.7%)	1161 (94.5%)	
Yes	33 (3.7%)	34 (10.3%)	67 (5.5%)	
**Chronic lung diseases**				**0.975**
No	844 (94.0%)	310 (93.9%)	1154 (94.0%)	
Yes	54 (6.0%)	20 (6.1%)	74 (6.0%)	
**Cardiovascular diseases**				**0.006**
No	887 (98.8%)	318 (96.4%)	1205 (98.1%)	
Yes	11 (1.2%)	12 (3.6%)	23 (1.9%)	
**Hypertension**				**0.006**
No	846 (94.2%)	296 (89.7%)	1142 (93.0%)	
Yes	52 (5.8%)	34 (10.3%)	86 (7.0%)	
**Diabetes**				**0.117**
No	858 (95.5%)	308 (93.3%)	1166 (95.0%)	

Yes	40 (4.5%)	22 (6.7%)	62 (5.0%)	
**Chronic liver diseases**				**0.8**
No	896 (99.8%)	329 (99.7%)	1225 (99.8%)	
Yes	2 (0.2%)	1 (0.3%)	3 (0.2%)	
**Autoimmune diseases**				**0.094**
No	896 (99.8%)	327 (99.1%)	1223 (99.6%)	
Yes	2 (0.2%)	3 (0.9%)	5 (0.4%)	
**Gestation trimester**				**0.29**
First trimester	4 (0.4%)	0 (0.0%)	4 (0.3%)	
Second trimester	11 (1.2%)	1 (0.3%)	12 (1.0%)	
Third trimester	4 (0.4%)	1 (0.3%)	5 (0.4%)	

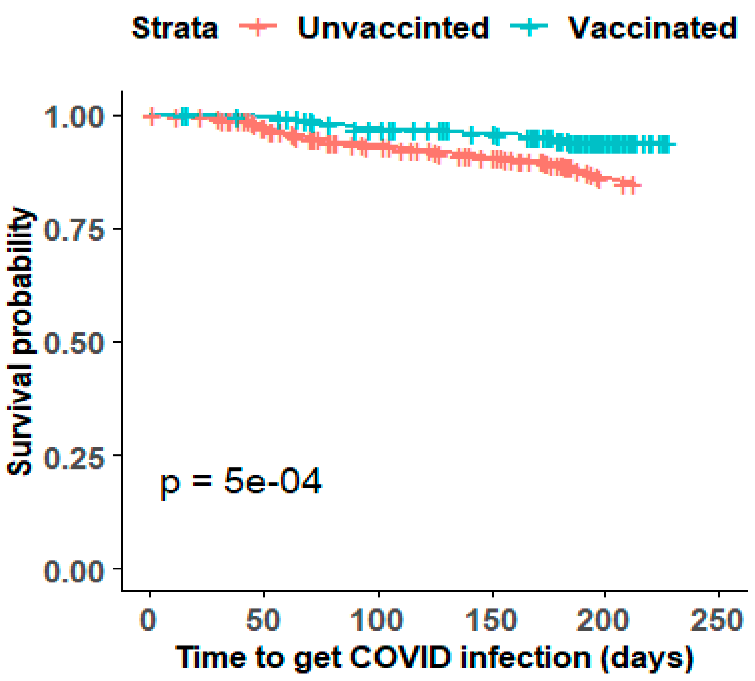

Figure S1. Kaplan-Meier curve for PCR confirmed COVID-19 cases.

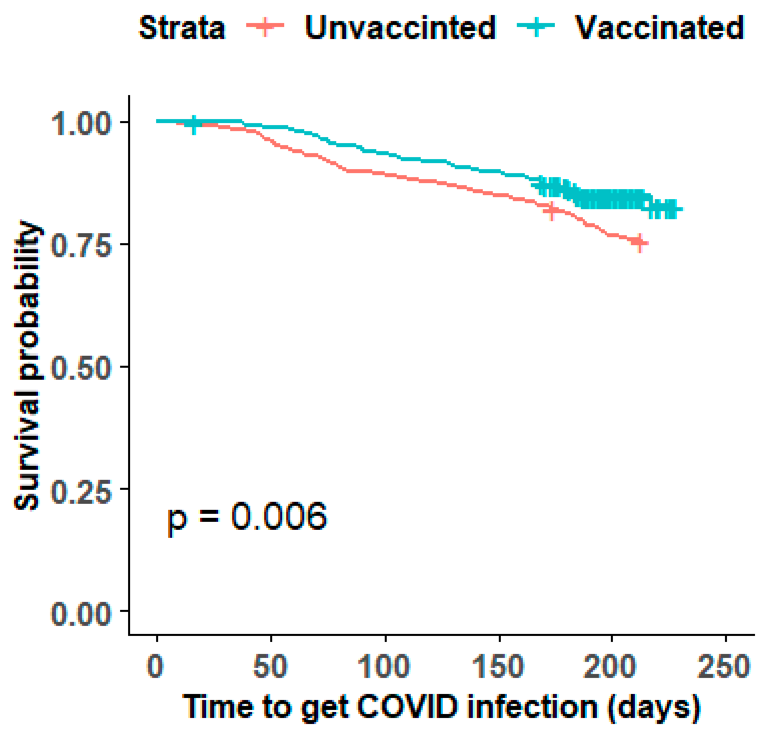

Figure S2. Kaplan-Meier curve for total COVID-19 cases (confirmed + suspected).

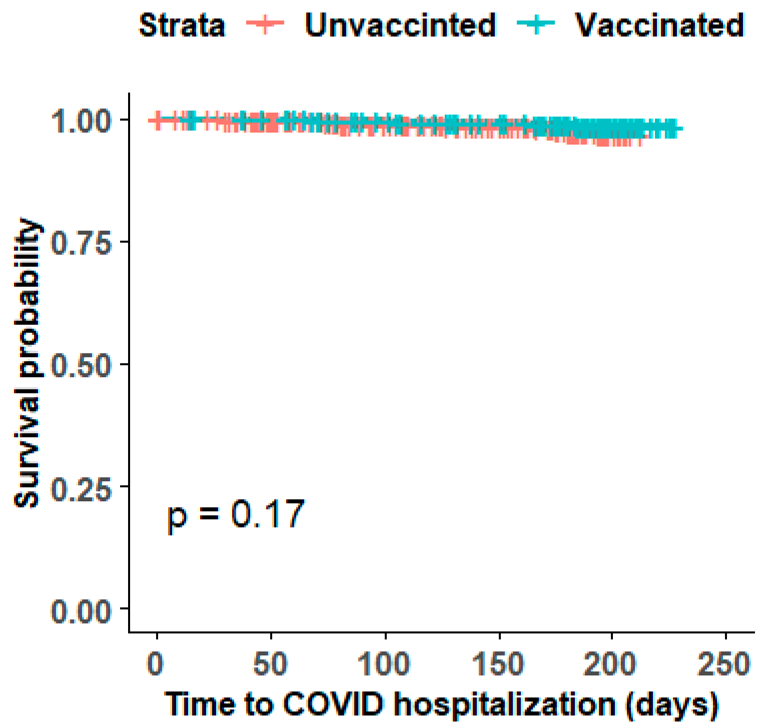

Figure S3. Kaplan-Meier curve for hospitalized COVID-19 cases.

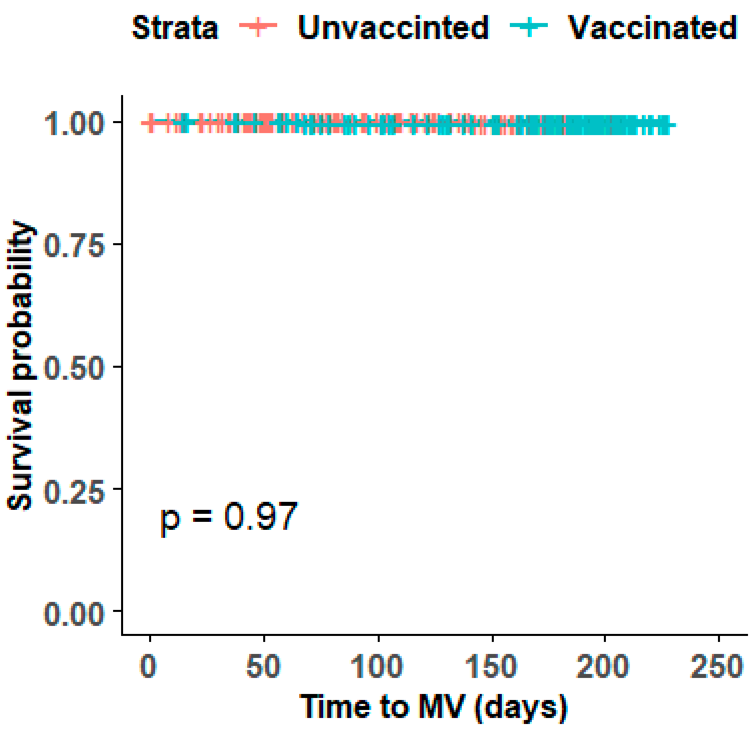

Figure S4. Kaplan-Meier curve for mechanically ventilated COVID cases.

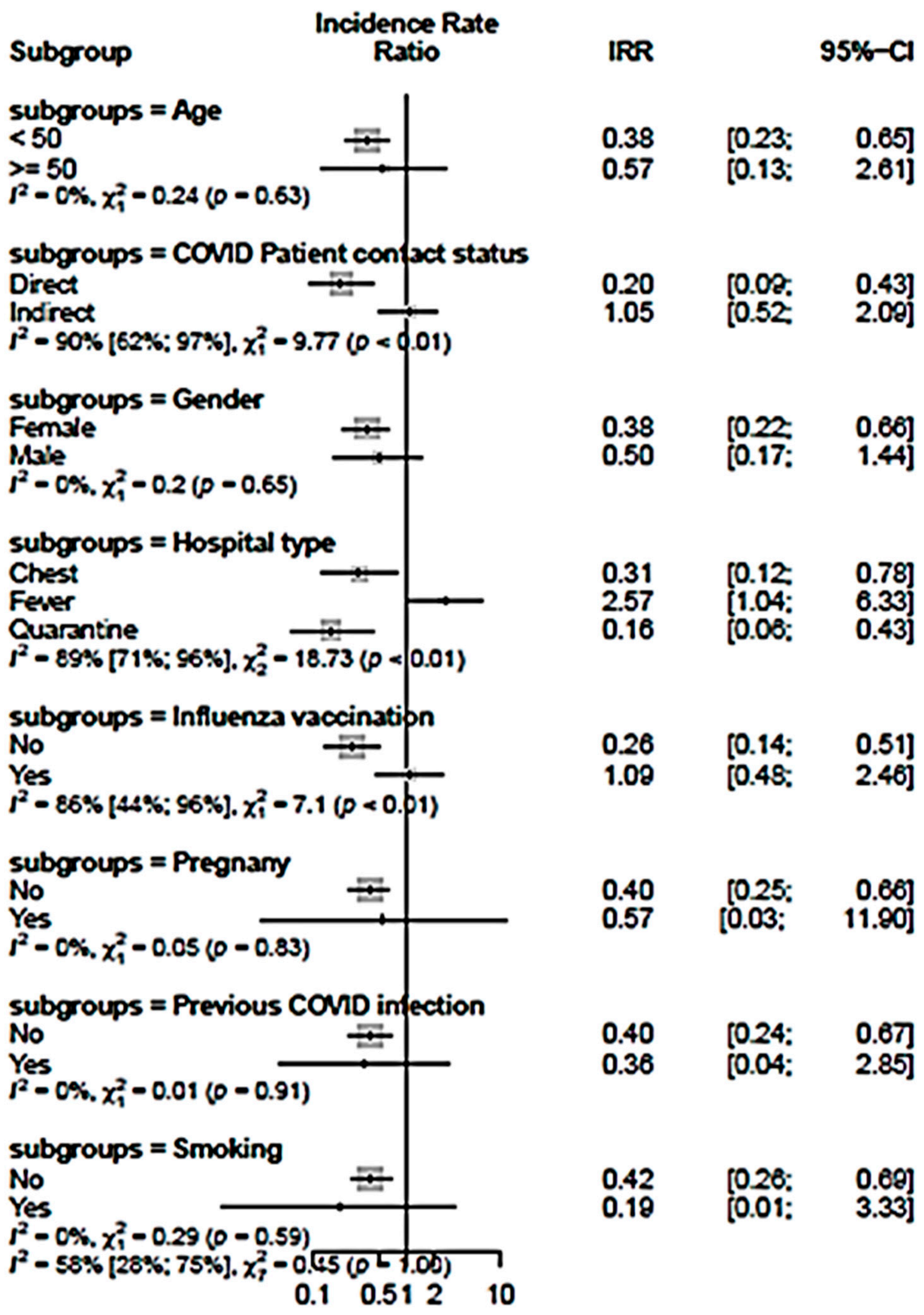

Figure S5. Incidence rate subgrouping of PCR-confirmed cases.

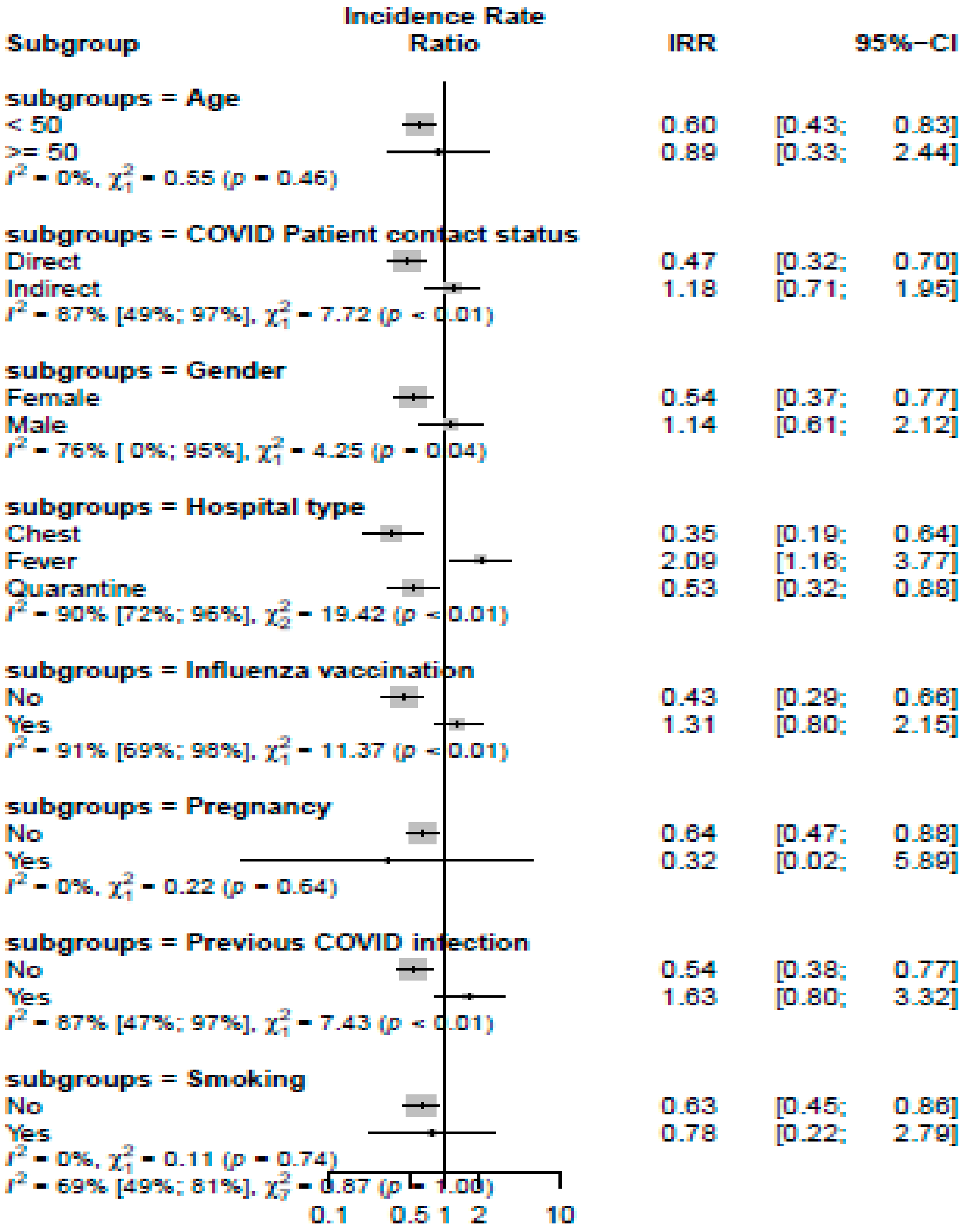

Figure S6. Incidence rate subgrouping for getting total COVID-19 infection (suspected + PCR confirmed)

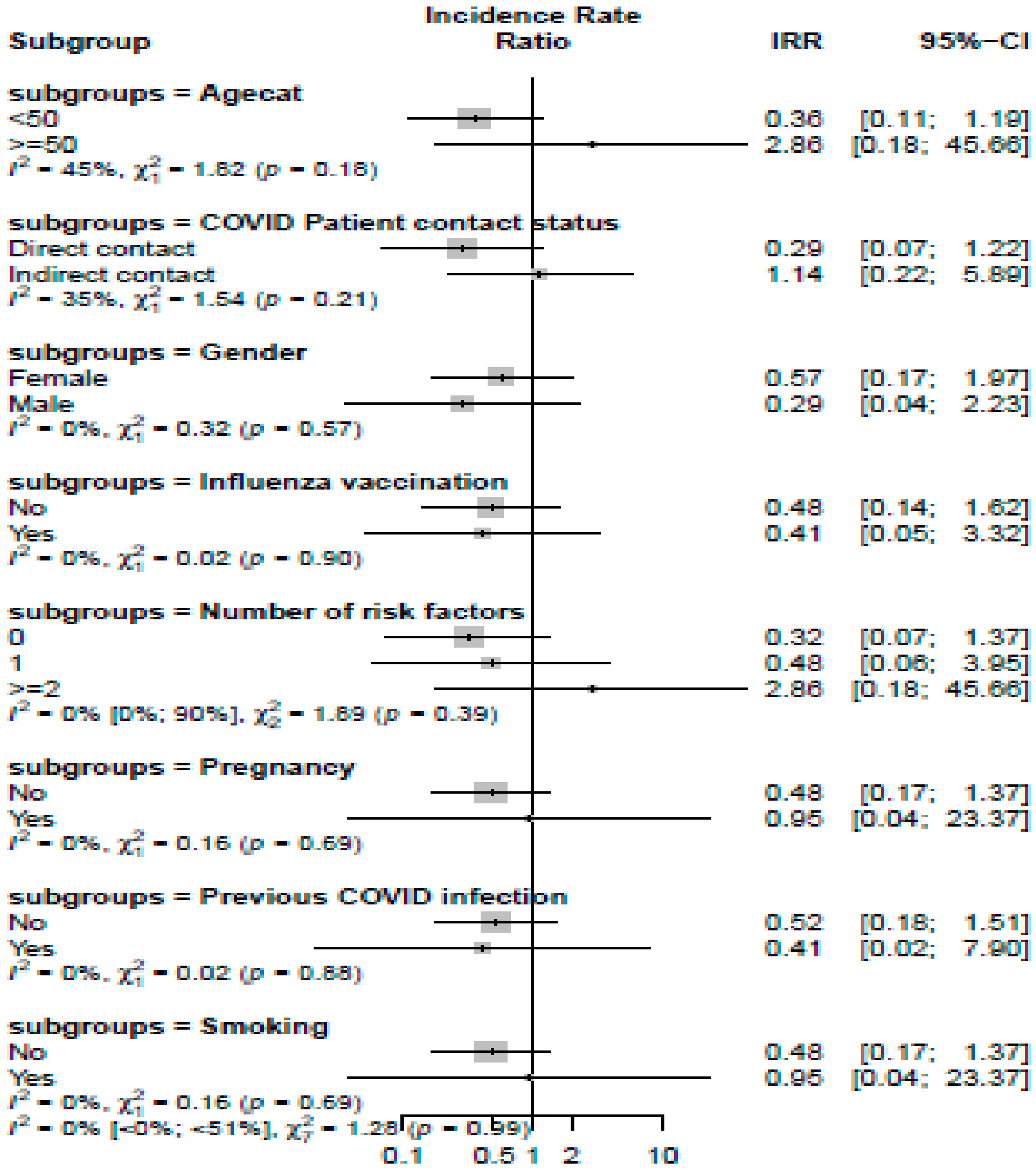

Figure S7. Incidence rate subgrouping for hospitalized COVID-19 infection (severe to critically ill)

Table S2. Reported side effects of vaccinated healthcare workers

	Both (N=51)	First dose only (N=120)	Second dose only (N=23)	No (N=135)	Total (N=329)
**Type of local side effects**					
No	17 (33.3%)		6 (26.1%)	135 (100%)	198 (60.2%)
Local pain	27 (52.9%)	63 (52.5%)	12 (52.2%)	0 (0%)	102 (31.0%)
Local pain, Erythema, Swelling	1 (2.0%)	3 (2.5%)	0 (0%)	0 (0%)	4 (1.2%)
local pain, Lymphadenopathy	1 (2.0%)	1 (0.8%)	0 (0%)	0 (0%)	2 (0.6%)
Numbness	1 (2.0%)	1 (0.8%)	0 (0%)	0 (0%)	2 (0.6%)
pain, Swelling	3 (5.9%)	4 (3.3%)	1 (4.3%)	0 (0%)	8 (2.4%)
Swelling	1 (2.0%)	0 (0%)	1 (4.3%)	0 (0%)	2 (0.6%)
Erythema	0 (0%)	2 (1.7%)	1 (4.3%)	0 (0%)	3 (0.9%)
Local pain, Erythema	0 (0%)	4 (3.3%)	1 (4.3%)	0 (0%)	5 (1.5%)
Local pain, rash	0 (0%)	2 (1.7%)	1 (4.3%)	0 (0%)	3 (0.9%)
**local side effects severity**					
grade 1 (mild)	29 (56.9%)	64 (53.3%)	16 (69.6%)	0 (0%)	109 (33.1%)
grade 2 (moderate)	5 (9.8%)	16 (13.3%)	1 (4.3%)	0 (0%)	22 (6.7%)
No	17 (33.3%)	40 (33.3%)	6 (26.1%)	135 (100%)	198 (60.2%)
**Fever**					
grade 1 (mild)	9 (17.6%)	41 (34.2%)	2 (8.7%)	0 (0%)	52 (15.8%)
grade 2 (moderate)	4 (7.8%)	3 (2.5%)	2 (8.7%)	0 (0%)	9 (2.7%)
No	38 (74.5%)	76 (63.3%)	19 (82.6%)	135 (100%)	268 (81.5%)
**Highest temperature**					
Mean (SD)	37.2 (0.459)	37.3 (0.503)	37.2 (0.485)	37.0 (0)	37.2 (0.403)
Median [Min, Max]	37.0 [37.0, 39.0]	37.0 [37.0, 38.6]	37.0 [37.0, 38.5]	37.0 [37.0, 37.0]	37.0 [37.0, 39.0]
**The fever lasts for days**					
1	4 (7.8%)	19 (15.8%)	1 (4.3%)	0 (0%)	24 (7.3%)
2	8 (15.7%)	20 (16.7%)	1 (4.3%)	0 (0%)	29 (8.8%)
3	1 (2.0%)	5 (4.2%)	2 (8.7%)	0 (0%)	8 (2.4%)
No	38 (74.5%)	76 (63.3%)	19 (82.6%)	135 (100%)	268 (81.5%)
**Blood pressure classification (ACC/ AHA)**					
Hypertension 2	3 (5.9%)	5 (4.2%)	2 (8.7%)	0 (0%)	10 (3.1%)
Hypertension 1	3 (5.9%)	1 (0.8%)	0 (0%)	0 (0%)	4 (1.2%)
Elevated	0 (0%)	2 (1.7%)	0 (0%)	0 (0%)	2 (0.6%)
Normal	44 (86.3%)	114 (95.0%)	21 (91.3%)	135 (100%)	314 (95.4%)
Hypotension (80/50)	1 (2.0%)	0 (0%)	0 (0%)	0 (0%)	1 (0.3%)
**SBP/DBP after vaccination**					
110/70	2 (3.9%)	3 (2.5%)	1 (4.3%)	0 (0%)	6 (1.8%)
120/80	42 (82.4%)	109 (90.8%)	20 (87.0%)	135 (100%)	306 (93.0%)
130/90	3 (5.9%)	1 (0.8%)	0 (0%)	0 (0%)	4 (1.2%)
140/90	2 (3.9%)	3 (2.5%)	1 (4.3%)	0 (0%)	6 (1.8%)
150/95	1 (2.0%)	0 (0%)	0 (0%)	0 (0%)	1 (0.3%)
80/50	1 (2.0%)	0 (0%)	0 (0%)	0 (0%)	1 (0.3%)
130/80	0 (0%)	2 (1.7%)	0 (0%)	0 (0%)	2 (0.6%)
140/95	0 (0%)	1 (0.8%)	0 (0%)	0 (0%)	1 (0.3%)
150/90	0 (0%)	0 (0%)	1 (4.3%)	0 (0%)	1 (0.3%)

**Blood pressure abnormality days**					
1	6 (11.8%)	3 (2.5%)	1 (4.3%)	0 (0%)	9 (2.7%)
2	1 (2.0%)	3 (2.5%)	1 (4.3%)	0 (0%)	5 (1.5%)
3	0 (0%)	1 (0.8%)	0 (0%)	0 (0%)	1 (0.3%)
No	44 (86.3%)	113 (94.2%)	21 (91.3%)	135 (100%)	313 (95.1%)
**Blood pressure management drugs**					
ACEIs	2 (3.9%)	2 (1.7%)	2 (8.7%)	0 (0%)	6 (1.8%)
CCBs	2 (3.9%)	2 (1.7%)	0 (0%)	0 (0%)	4 (1.2%)
BB	0 (0%)	1 (0.8%)	0 (0%)	0 (0%)	1 (0.3%)
No	47 (92.2%)	115 (95.8%)	21 (91.3%)	135 (100%)	318 (96.7%)
**Myalgia**					
grade 1 (mild)	16 (31.4%)	34 (28.3%)	7 (30.4%)	0 (0%)	57 (17.3%)
grade 2 (moderate)	5 (9.8%)	2 (1.7%)	2 (8.7%)	0 (0%)	9 (2.7%)
grade 3 (severe)	1 (2.0%)	0 (0%)	1 (4.3%)	0 (0%)	2 (0.6%)
No	29 (56.9%)	84 (70.0%)	13 (56.5%)	135 (100%)	261 (79.3%)
**Headache**					
grade 1 (mild)	7 (13.7%)	24 (20.0%)	4 (17.4%)	0 (0%)	35 (10.6%)
grade 2 (moderate)	7 (13.7%)	11 (9.2%)	5 (21.7%)	0 (0%)	23 (7.0%)
grade 3 (severe)	0 (0%)	1 (0.8%)	0 (0%)	0 (0%)	1 (0.3%)
No	37 (72.5%)	84 (70.0%)	14 (60.9%)	135 (100%)	270 (82.1%)
**Headache days**					
0.5	2 (3.9%)	0 (0%)	0 (0%)	0 (0%)	2 (0.6%)
1	4 (7.8%)	17 (14.2%)	2 (8.7%)	0 (0%)	23 (7.0%)
2	7 (13.7%)	16 (13.3%)	4 (17.4%)	0 (0%)	27 (8.2%)
3	1 (2.0%)	3 (2.5%)	1 (4.3%)	0 (0%)	5 (1.5%)
4	0 (0%)	0 (0%)	2 (8.7%)	0 (0%)	2 (0.6%)
No	37 (72.5%)	84 (70.0%)	14 (60.9%)	135 (100%)	270 (82.1%)
**Nausea/vomiting**					
grade 1 (mild)	1 (2.0%)	4 (3.3%)	1 (4.3%)	0 (0%)	6 (1.8%)
grade 2 (moderate)	1 (2.0%)	1 (0.8%)	0 (0%)	0 (0%)	2 (0.6%)
No	49 (96.1%)	115 (95.8%)	22 (95.7%)	135 (100%)	321 (97.6%)
**Nausea/vomiting days**					
0.5	0 (0%)	1 (0.8%)	0 (0%)	0 (0%)	1 (0.3%)
1	0 (0%)	2 (1.7%)	0 (0%)	0 (0%)	2 (0.6%)
2	2 (3.9%)	1 (0.8%)	1 (4.3%)	0 (0%)	4 (1.2%)
3	0 (0%)	1 (0.8%)	0 (0%)	0 (0%)	1 (0.3%)
No	49 (96.1%)	115 (95.8%)	22 (95.7%)	135 (100%)	321 (97.6%)
**Arthralgia**					
grade 1 (mild)	6 (11.8%)	12 (10.0%)	4 (17.4%)	0 (0%)	22 (6.7%)
grade 2 (moderate)	2 (3.9%)	1 (0.8%)	2 (8.7%)	0 (0%)	5 (1.5%)
No	43 (84.3%)	107 (89.2%)	17 (73.9%)	135 (100%)	302 (91.8%)
**Fatigue**					
grade 1 (mild)	15 (29.4%)	34 (28.3%)	2 (8.7%)	0 (0%)	51 (15.5%)
grade 2 (moderate)	9 (17.6%)	14 (11.7%)	4 (17.4%)	0 (0%)	27 (8.2%)
grade 3 (severe)	0 (0%)	3 (2.5%)	1 (4.3%)	0 (0%)	4 (1.2%)
No	27 (52.9%)	69 (57.5%)	16 (69.6%)	135 (100%)	247 (75.1%)
**Chills**					
grade 1 (mild)	4 (7.8%)	10 (8.3%)	4 (17.4%)	0 (0%)	18 (5.5%)
grade 2 (moderate)	1 (2.0%)	1 (0.8%)	0 (0%)	0 (0%)	2 (0.6%)

No	46 (90.2%)	109 (90.8%)	19 (82.6%)	135 (100%)	309 (93.9%)
**Diarrhea**					
grade 1 (mild)	3 (5.9%)	1 (0.8%)	2 (8.7%)	0 (0%)	6 (1.8%)
No	48 (94.1%)	119 (99.2%)	21 (91.3%)	135 (100%)	323 (98.2%)
**Other adverse event if present**					
Backache	1 (2.0%)	0 (0%)	0 (0%)	0 (0%)	1 (0.3%)
Eye pain	1 (2.0%)	0 (0%)	0 (0%)	0 (0%)	1 (0.3%)
Leg muscle twitches	1 (2.0%)	0 (0%)	0 (0%)	0 (0%)	1 (0.3%)
Difficulty in breathing	0 (0%)	1 (0.8%)	0 (0%)	0 (0%)	1 (0.3%)
hyperpigmentation at face	0 (0%)	1 (0.8%)	0 (0%)	0 (0%)	1 (0.3%)
Mild Edema face hand use avil and dexamethasone amp	0 (0%)	1 (0.8%)	0 (0%)	0 (0%)	1 (0.3%)
Rhinorrhea	0 (0%)	1 (0.8%)	0 (0%)	0 (0%)	1 (0.3%)
Runny nose and cough	0 (0%)	1 (0.8%)	0 (0%)	0 (0%)	1 (0.3%)
Sore throat	0 (0%)	1 (0.8%)	0 (0%)	0 (0%)	1 (0.3%)
insomnia	0 (0%)	0 (0%)	1 (4.3%)	0 (0%)	1 (0.3%)
**Duration of side effects (days)**					
Mean (SD)	2.86 (3.24)	18.0 (46.5)	2.67 (0.577)	NA (NA)	10.4 (33.0)
Median [Min, Max]	2.00 [1.00, 10.0]	2.50 [1.00, 150]	3.00 [2.00, 3.00]	NA [NA, NA]	2.00 [1.00, 150]
**Management of adverse effect**					
No treatment	35 (68.6%)	71 (59.2%)	19 (82.6%)	135 (100%)	260 (79.0%)
Foradil and meflonide inhalers, paracetamol	1 (2.0%)	0 (0%)	0 (0%)	0 (0%)	1 (0.3%)
NSAIDS	1 (2.0%)	4 (3.3%)	1 (4.3%)	0 (0%)	6 (1.8%)
Paracetamol	14 (27.5%)	42 (35.0%)	3 (13.0%)	0 (0%)	59 (17.9%)
Antihistamines	0 (0%)	1 (0.8%)	0 (0%)	0 (0%)	1 (0.3%)
Nasal decongestants +Paracetamol	0 (0%)	1 (0.8%)	0 (0%)	0 (0%)	1 (0.3%)
Paracetamol + antihistamines	0 (0%)	1 (0.8%)	0 (0%)	0 (0%)	1 (0.3%)
